# An Overview on the Use of Extracts from Medicinal and Aromatic Plants to Improve Nutritional Value and Oxidative Stability of Vegetable Oils

**DOI:** 10.3390/foods11203258

**Published:** 2022-10-18

**Authors:** Saïd Gharby, Samira Oubannin, Hasna Ait Bouzid, Laila Bijla, Mohamed Ibourki, Jamila Gagour, Jamal Koubachi, El Hassan Sakar, Khalid Majourhat, Learn-Han Lee, Hicham Harhar, Abdelhakim Bouyahya

**Affiliations:** 1 Biotechnology, Analytical Sciences and Quality Control Team, Polydisciplinary Faculty of Taroudant, Ibn Zohr University, Agadir 80000, Morocco; 2 African Sustainable Agriculture Research Institute (ASARI), Mohammed VI Polytechnic University (UM6P), Laayoune 70000, Morocco; 3 Laboratory of Biology, Ecology and Health, FS, Abdelmalek Essaadi University, Tetouan 93002, Morocco; 4 Novel Bacteria and Drug Discovery Research Group (NBDD), Microbiome and Bioresource Research Strength (MBRS), Jeffrey Cheah School of Medicine and Health Sciences, Monash University Malaysia, Bandar Sunway, Subang Jaya 47500, Selangor, Malaysia; 5 Laboratory of Materials, Nanotechnology and Environment LMNE, Mohammed V University in Rabat, Rabat 10100, Morocco; 6 Laboratory of Human Pathologies Biology, Department of Biology, Genomic Center of Human Pathologies, Faculty of Sciences, Mohammed V University in Rabat, Rabat 10100, Morocco

**Keywords:** enrichment, extraction, natural antioxidants, oxidative stability, vegetable oils

## Abstract

Oil oxidation is the main factor limiting vegetable oils’ quality during storage, as it leads to the deterioration of oil’s nutritional quality and gives rise to disagreeable flavors. These changes make fat-containing foods less acceptable to consumers. To deal with this problem and to meet consumer demand for natural foods, vegetable oil fabricators and the food industry are looking for alternatives to synthetic antioxidants to protect oils from oxidation. In this context, natural antioxidant compounds extracted from different parts (leaves, roots, flowers, and seeds) of medicinal and aromatic plants (MAPs) could be used as a promising and sustainable solution to protect consumers’ health. The objective of this review was to compile published literature regarding the extraction of bioactive compounds from MAPs as well as different methods of vegetable oils enrichment. In fact, this review uses a multidisciplinary approach and offers an updated overview of the technological, sustainability, chemical and safety aspects related to the protection of oils.

## 1. Introduction

Vegetable oils (VOs) consumption has increased worldwide due to its health benefits [1]. Triacylglycerols are the main constituents of lipids (~up to 99%) and important storage lipids. These triglycerides are composed of a glycerol unit esterified by three fatty acids whose proportions depend on the plant from which they are derived (soybean, olive, argan, rapeseed, sunflower, peanut, etc.) [2,3]. Along with triglycerides, there is a very important minority fraction (unsaponifiable matter, at least ~1%) namely polyphenols, phytosterols, minerals, vitamins, resinous esters, etc. [4].

Thanks to this biochemical composition, vegetable oils play a very important role as a source of energy for human metabolic processes, essential fatty acids, tocopherols, and fat-soluble vitamins, as well as a structural role [5,6]. Oxidation is principally responsible for the quality deterioration of industrial oils, causing loss of nutritional value and giving rise to disagreeable odors. These make VOs and food containing VOs less acceptable to consumers [7]. Furthermore, lipid oxidation leads to certain toxic compounds, such as reactive carbonyl compounds, which, in turn, generate progressive lipid peroxidation and products. These are possibly unsafe for human health [7].

In addition, lipid oxidation level depends on a set of factors internal to VOs. Among them, are the degree of unsaturation, the presence of antioxidant compounds (tocopherols, polyphenols, etc.), and metals such as copper and iron. It depends also on oils’ external factors including storage conditions (availability of dioxygen, temperature, and light exposure) [8,9].

Many approaches can be used to enhance the stability oxidative of the oil throughout processing and storage. Among them the prevention of light, dioxygen, and high temperatures optimize oil extraction conditions to improve the content of bioactive compounds and antioxidant compounds [10].

Along with natural antioxidant compounds present in oils. Oil oxidative stability and quality can be improved using synthetic antioxidant compounds. Among these, butylated hydroxyanisole (BHA), butylated hydroxytoluene (BHT), tertbutyl hydroquinone (TBHQ), and propyl gallate (PG) are the most widely used. Although, there are doubts about their health effects, and some evidence highlights the carcinogenic effects of these synthetic compounds [7,11].

For this reason, other alternatives for enriching VOs have been proposed. In particular, the use of natural antioxidants from secondary streams, food by-products, and agri-food wastes. especially as food processing waste represents 30.6 million tonnes per year, 35% of which come from fruit and vegetables [12]. However, these by-products require processing, involving the addition of chemicals (solvents), so in addition to the extraction of phenolic compounds. It is very likely that there will be an extraction of other undesirable compounds making it necessary to look for more natural sources of antioxidants.

On the other hand, medicinal and aromatic plants (MAPs) seem to be a good target for natural antioxidant compound extraction. Their use, since ancient times, by several civilizations for their numerous health and curative properties [13]. Several studies were devoted to the enrichment of oils with antioxidants from MAPs [14,15,16,17,18].

This review focuses on the enrichment of VOs with bioactive compounds from MAPs to protect VOs from oxidation in the first instance. Based on a quantitative and qualitative analysis of published works on the use of natural antioxidants to improve VOs’ oxidative stability. This review highlights the role of antioxidants in improving the oxidative stability of VOs, concentrating on their mode of action as well as the extraction methods and their impacts in recovering antioxidants from MAPs as well as enrichment methods used to evaluate the oxidative stability and efficacy of antioxidants.

## 2. Bibliometric Analysis

### 2.1. Database Choice

**Figure 1:**
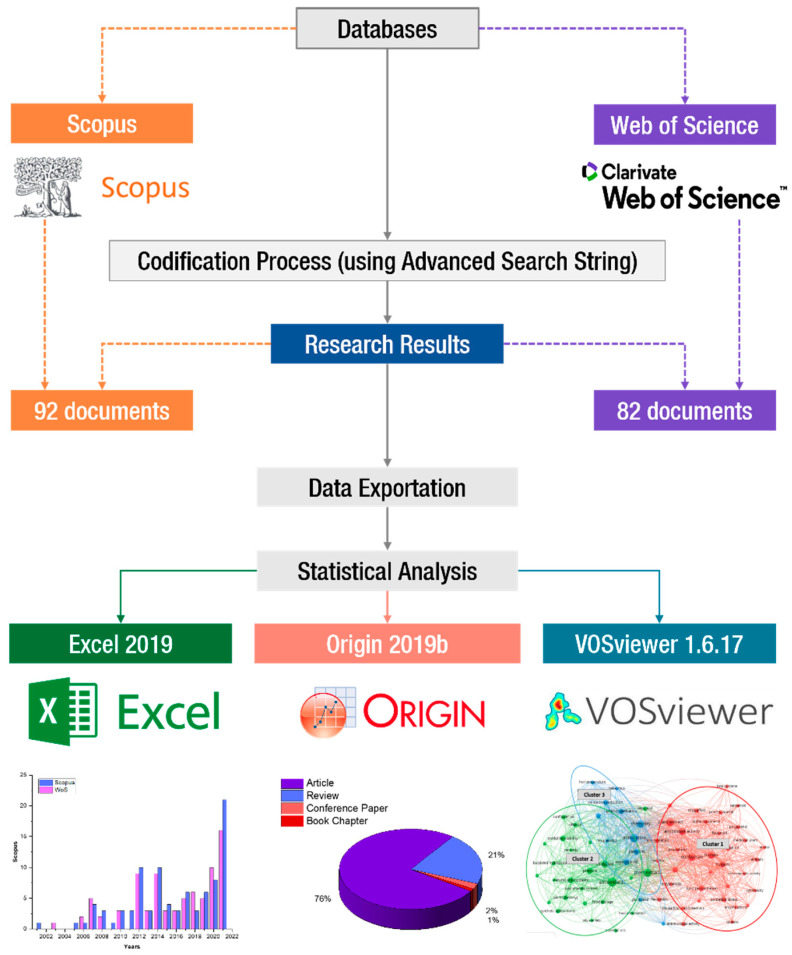
A scheme of bibliometric analysis methodology.

One of the most important steps in a bibliometric analysis is to select the appropriate databases that are relevant to the research purpose (Figure 1). Bibliometric analysis is restricted by the type of available information [19]. Thus, the information sources have to be trusted and adapted to conduct such an analysis and provide the most efficient decisions [20]. The Scopus and Web of Science (WoS) databases are both currently accessible, and their utilization in literature is consequently widespread [19].

### 2.2. Indicators

**Figure 2:**
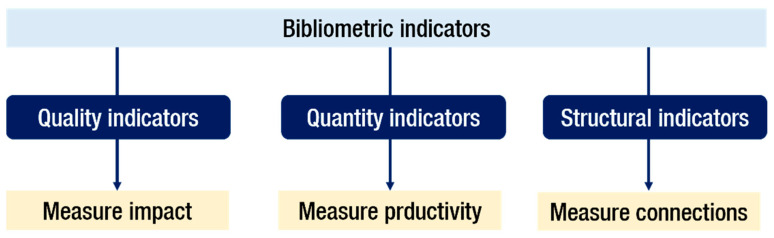
Types of bibliometric indicators.

After selecting the databases, the second step is to identify the appropriate indicators to evaluate the resulting sample. The published papers present various types of bibliometric indicators [21]. According to Gema et al. [19], there are three different types of bibliometric indicators: (i) quality indicators (to measure impact); (ii) quantity indicators (to measure productivity), and (iii) structural indicators (to measure connections) (Figure 2).

### 2.3. Advanced Research (2000 Onward)

This review investigates the research areas of VOs oxidation along with MAPs’ antioxidants. Our study was carried out in March 2022 using the Scopus and WoS databases. It examines scientific research studies from 2000 onward. The date of publication of the first paper on improving the oxidative stability of VO using plant extracts is listed in both databases (Scopus and WoS). This bibliometric analysis covers the most common fields of knowledge, the journals with the most publications, the most prolific authors, the most productive countries, the most cited studies, publications trends, document types, and countries or territories of origin. To conduct our study, the advanced search string used for Scopus database is (TITLE-ABS-KEY-AUTH((“enriche*” OR “enhanc*” OR “improve*” OR “amelior*”) AND (“oxidative stability” OR “*oxidation*” OR “shelf-life*” OR “shelf life*”) AND (“*phenol*” OR “antioxida*”) AND (“natural*”) AND (“extract*”) AND (“oil*”) AND (“aromatic and medicinal plant*” OR “medicinal and aromatic plant*” OR “AMP*” OR “MAP*” OR “plant*”)) AND NOT TITLE-ABS-KEY (“meat*”) AND NOT TITLE-ABS-KEY (“mustard”) AND NOT TITLE-ABS-KEY (“rat*”) AND NOT TITLE-ABS-KEY (“packaging”) AND NOT TITLE-ABS-KEY(“cheese*”)AND NOT TITLE-ABS-KEY (“lamb?”) AND NOT TITLE-ABS-KEY (“sausage*”) AND NOT TITLE-ABS-KEY (“International Multidisciplinary Scientific Geoconference SGEM”) AND NOT TITLE-ABS- KEY (“silver”) AND NOT TITLE-ABS-KEY (“hamburger*”) AND NOT TITLE-ABS-KEY(“mice”) AND NOT TITLE-ABS-KEY(“mouse”) AND NOT TITLE-ABS-KEY(“potato”) AND NOT (“biodiesel”) AND NOT TITLE-ABS-KEY(“germination”) AND NOT TITLE-ABS-KEY(“emulsion*”) AND NOT TITLE-ABS-KEY(“rice”) AND NOT TITLE-ABS-KEY(“fish oil”) AND PUBYEAR < 2022 AND PUBYEAR > 1999).

However, for the WoS database, we have used the following string, (TS = (“enriche*”) OR TS = (“enhanc*”) OR TS = (“improve*”) OR TS = (“amelior*”)) AND (TS = (“oxidative stability”) OR TS = (“*oxidation*”) OR TS = (“shelf-life*”) OR TS = (“shelf life*”)) AND (TS = (“*phenol*”) OR TS = (“antioxida*”)) AND TS = (“natural*”) AND TS = (“extract*”) AND TS = (“oil*”) AND (TS = (“aromatic and medicinal plant*”) OR TS = (“medicinal and aromatic plant*”) OR TS = (“AMP*”) OR TS = (“MAP*”) OR TS = (“plant*”)) NOT TS = (“meat*”) NOT TS = (“mustard”) NOT TS = (“rat*”) NOT TS = (“packaging”) NOT TS = (“cheese*”) NOT TS = (“lamb?”) NOT TS = (“sausage*”) NOT TS = (“International Multidisciplinary Scientific Geoconference SGEM”) NOT TS = (“silver”) NOT TS = (“mice”) NOT TS = (“mouse”) NOT TS = (“potato”) NOT TS = (“biodiesel”) NOT TS = (“germination”) NOT TS = (“emulsion*”) NOT TS = (“rice”) NOT TS = (“fish oil”)) AND PY = (2000–2021). After, the outcomes were exported as an excel file according to the used indicators.

### 2.4. Analysis

**Figure 3:**
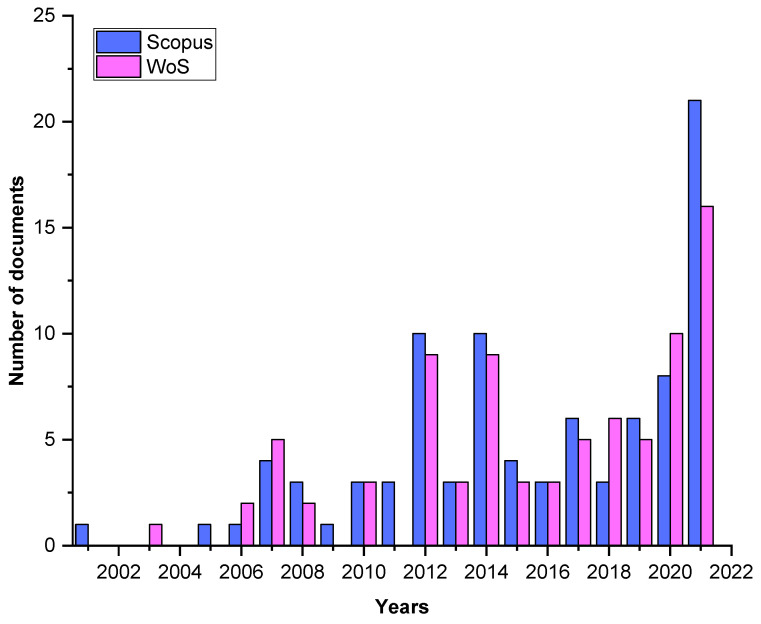
Publications trend of enrichment of VOs with plants antioxidants (based on data retrieved from Scopus and WoS databases).

The research resulted in 92 and 82 publications in Scopus and WoS databases, respectively (Figure 3). The most cited paper (157 times) was that of Bouaziz et al. [22], published in 2008 in Food Chemistry, addressing the effect of storage on refined and husk olive oils composition and their stabilization by using natural antioxidants from ‘Chemlali’ olive leaves.

**Figure 4:**
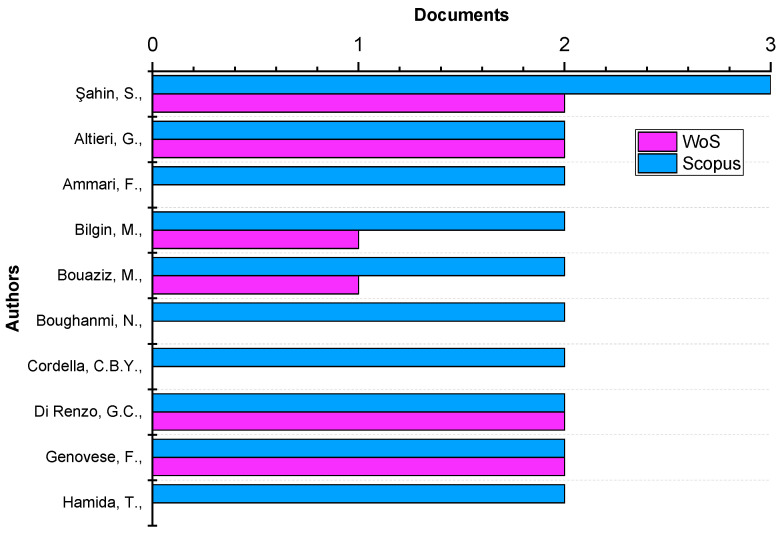
Most productive authors (based on data from Scopus and WoS).

The most prolific author was Şahin [23] with 3 documents (Figure 4). Her most cited paper (17 times) dealt with the impact of olive extracts containing oleuropein on the quality of virgin olive oil [23].

Among the most cited publications, the work of Kammerer et al. [24] described the recovery of polyphenols from plant food processing by-products and their application as valuable food ingredients. Another one, published in the same year, is focused on the effect of natural antioxidants extracted from animal and vegetable resources on the oxidative stability of soybean oil [25].

**Figure 5:**
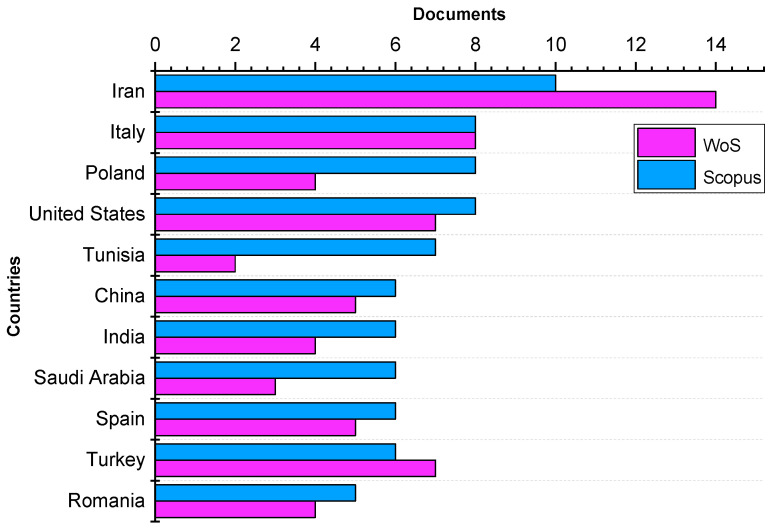
Most productive countries (according to WoS and scopus data).

Figure 5 shows the most productive countries or territories. According to Scopus and WoS databases, Iran was the most productive country with 10 and 14 publications, respectively, followed by Italy, Poland, the United States, and Tunisia. The main keywords covered for documents reported from Iran are antioxidant(s), antioxidant activities, lipid peroxidation, oxidative stability, and extraction. One of the most productive institutions was the Gorgan University of Agricultural Sciences and Natural Resources, with 3 documents. The most cited paper was that of Taghvaei et al. [25], which was formerly described. However, another work reported by Hosseini and Jafari [26], published in Advances in Colloid and Interface Science, introduces nano/microencapsulated bioactive ingredients for prolonging the shelf-life of food products.

**Figure 6:**
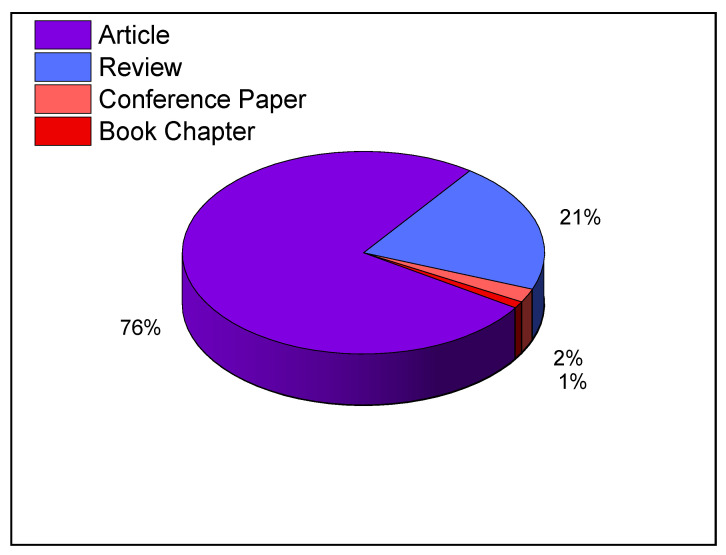
Distribution of documents by type (based on data from Scopus).

Figure 6 reports the distribution of documents by type according to the Scopus database. It includes mainly “research article” with 76%, followed by “review article” with 21%, and “conference paper” with 2%.

Table 1 represents the most productive journals in the enrichment of VOs with natural antioxidants. According to the Scopus database, a total of 79 journals have published research studies on enhancing the oxidative stability of VOs using plants between the years 2000 and 2021. In this section, we have focused on journals with a minimum of 2 documents. There were only 11 journals that met the threshold, with the Journal of Food Science, hosted by the Wiley-Blackwell publisher, reporting the highest number of papers, research on oils enrichment with MAPs’ extracts with 6 outputs. Followed by the Journal of Agricultural and Food Chemistry with 4 documents. The remaining journals include the International Journal of Molecular Sciences, Food Chemistry, Journal of Food Processing and Preservation, and Journal of Food Science and Technology, with 3 publications each.

Of these 6 described journals, two are published by Wiley-Blackwell, while the Journal of Agricultural and Food Chemistry is published by the American Chemical Society, and Food Chemistry is hosted on Elsevier. Citations per journal can serve as a strong index of influence and reach [27]. In terms of average citation per journal, the Journal of Food Chemistry came out on top (109.33), followed by Food Research International and International Journal of Molecular Sciences with 58 and 40.33, respectively (Table 1). Of 11 journals represented in Table 1, four are in the Elsevier database, and all journals address aspects of agricultural and biological sciences.

**Figure 7:**
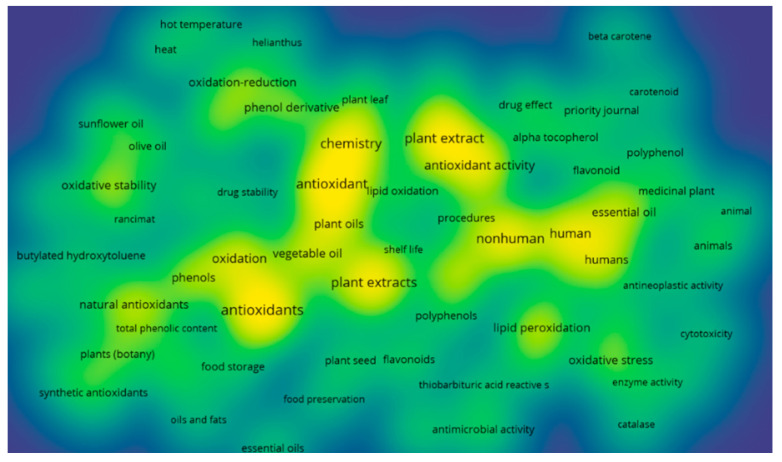
Density map of clusters based on Scopus database performed via VOSviewer software.

**Figure 8:**
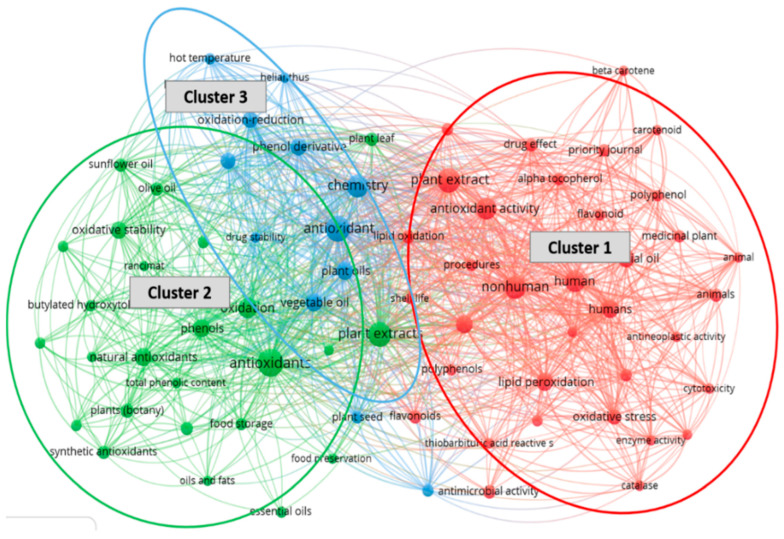
Terms map based on Scopus database carried out using VOS viewer software.

The bibliometric analysis software VOSviewer version 1.6.17 (Leiden, Netherlands) was selected for this study instead of other similar ones, principally due to its professional efficiency in analyzing clustered search results [28,29]. It examines words/terms contained in abstracts and titles of published papers by splitting paragraphs into separate words and phrases, connecting them to the citation data of the relevant papers. The results are presented as a density map or term bubble map with default settings [7] (Figure 7 and Figure 8).

To facilitate the bubble map, words/terms occurring at a minimum of 5 times in the publications were examined and visualized. Of the 1647 keywords, 76 met the selected threshold, and 3 of them were manually eliminated. The obtained results are presented in the form of network visualization and density diagrams. According to the terms and density maps (Figure 7 and Figure 8), three clusters are generated. Cluster 1 (35 items) includes such terms as “alpha tocopherol”, “antioxidant activity”, “plant extract”, “lipid oxidation”, “polyphenol”, “beta carotene”, and “flavonoid”. Cluster 2 (25 items) contains such keywords as “food preservation”, “food storage”, “olive oil”, “sunflower oil”, and “vegetable oils”. Cluster 3 (13 items) consists of the following keywords “chemistry”, “hot temperature”, “heat,” “oxidation reduction”, “quercetin”, and “seed kernel.” Cluster 6 (4 items) includes four keywords: corrosion, enzyme inhibition, inhibition, and steel.

## 3. Vegetable Oils Oxidation

**Figure 9:**
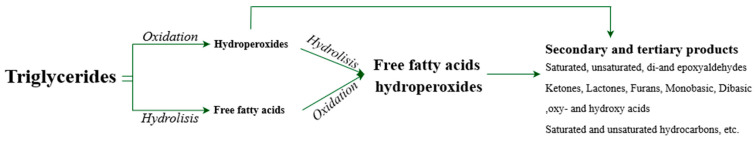
Oxidative and hydrolytic degradation reaction pathways in VOs.

VOs and fats are important constituents of human consumption and are essential components of our daily diet [30]. They are classified as vegetable or animal oils and fats depending on their source. VOs represented the major part of the world’s production in 2020/2021. In fact, VOs global production is 207 million metric tons [31]. The four major VOs are palm, soybean, rapeseed, and sunflower [32] VOs obtained by solvent extraction or mechanical expelling of oleaginous seeds (sunflower, soybeans, rapeseed, etc.) or oleaginous fruit like olive and palm [30]. VOs quality is defined by both organoleptic and compositional properties. These determine also agro-industrial preferences and consumer acceptance [7]. The occurrence of off-odors and off-flavors in oils/fats is usually associated with oxidative and/or hydrolytic degradations of triglycerides, and these major degradation reactions are presented in Figure 9.

**Figure 10:**
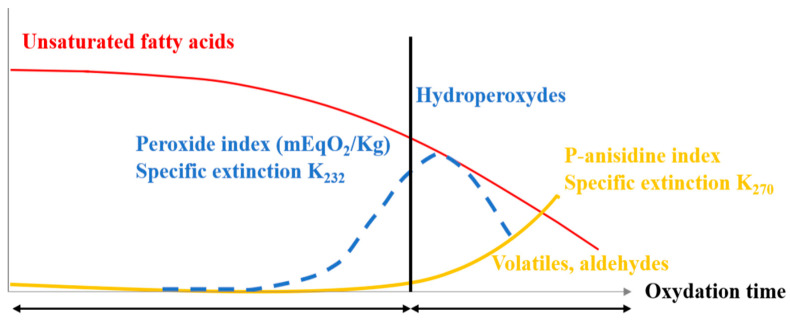
Kinetics of unsaturated fatty acids degradation.

Several studies have demonstrated that the principal quality attributes in VOs are their oxidative stability. Indeed, the VOs oxidation (Figure 9) is a complex series of reactions that result in smells, rancidity, and off-flavors [33]. This phenomenon is an important factor responsible for VO quality during the storage and production process. It is also the most important and prominent deleterious process in oils, which is related to the final nutritional and sensory quality [34]. Therefore, the shelf life of VOs depends on their oxidative stability [35,36]. Moreover, it is remarkable that throughout the oxidation process, some toxic compounds, such as reactive carbonyl compounds can result in advanced lipid peroxidation end products. These are possibly dangerous to human health [37]. Many factors can contribute to the oil oxidation, including high temperature, storage conditions, high dioxygen availability, fatty acids composition, and their level of polyunsaturated, as well as the existence of pro-oxidants such as chlorophylls, heavy metals, and metal ions (Fe^3+^ and Cu^2+^), [38,39]. The oil oxidation reactions are explained by the conventional free radical chain phenomenon, which begins with radical reactions on unsaturated fatty acids [40]. These reactions consist of three stages namely initiation followed by propagation, and termination [41]. In fact, the initiation stage involves the monomolecular phase of hydroperoxide generation along with peroxyl radical scavenging via antioxidants [42]. Whereas, the propagation stage consists of autocatalytic, monomolecular, and bimolecular reactions [43]. Nevertheless, the termination stage is mainly defined by the decomposition of hydroperoxides on one hand and the increased formation of secondary oxidation products on the other hand [41], as evidenced in Figure 10. The oxidation mechanism is summarized in the following reactions [44]:

Initiation phase:

RH + O_2_ → R^•^ + HOO^•^

Propagation phase:

R^•^ + O_2_ → ROO^•^ ROO^•^ + RH → ROOH + R^•^

ROOH → RO^•^ + ^•^OH

RO^•^ + RH → ROH + R^•^

^•^OH + RH → H_2_O + R^•^

Termination phase:

R^•^ + ^•^R → R-R

ROO^•^ + ROO^•^ → ROOOOR

Oxidative stability depends upon the balance of various extrinsic and intrinsic factors. Among them are fatty acids unsaturation, environment conditions, composition of minor components, delivery techniques, and use of antioxidants. Lipid oxidation induces negative effects on human health and also food quality. Therefore, efforts should be made to reduce oxidation and enhance the oxidative stability of lipid products [45].

Improving the oxidative stability of VO is an excellent way to extend the shelf life of oils. It allows also to reduce the content of off-flavors [10]. The various techniques and methods used to enhance the stability of VOs and improve oxidative stability are well documented. Firstly, modification of the fatty acid composition by natural selection of plants modification is a possible way to improve the oxidative stability of VOs [46]. Secondly, blending VOs with different fatty acid compositions is suggested as one of the simplest methods to improve both the quality and stability of VOs [47]. Thirdly, other authors have reported that the processing technique can improve VO oxidative stability. Indeed, Matthäus (2012), has mentioned that the virgin oils obtained by cold pressing are extremely popular due to their typical color, taste, and flavor, and are very rich in natural antioxidants [48]. In addition to this, roasting is a positive treatment that has a strong impact on VO quality with regard to appearance flavor, color, texture, and also the stability [49], especially in virgin oils like argan [50], cactus [49], and sesame oils [51]. Finally, many industries use synthetic or natural antioxidants, to improve VO stability, especially those which are refined. However, several scientific studies associated synthetic antioxidants with health risks due to their carcinogenesis effects [7]. Recently, various new studies develop and validate safe and sustainable methods, alternatives to synthetic antioxidants, to enhance VOs’ oxidative stability with the use of extracts obtained, via green methods, from MAPs, food industry side streams, and by-products [7].

## 4. Antioxidants: A Masterpiece of Mother Nature

One of the main problems in food preservation is rancidity, which is linked partially to the oxidation of unsaturated fatty acids in edible oils during processing, transportation, storage, and final preparation of edible Vos [52,53]. Many methods have been developed in order to control the rate and extent of lipid oxidation in foods, and then ensure the proper preservation of food products containing fats, which remains a fundamental objective for the food industry [54]. Antioxidants can be used as an option to have a longer shelf life of fatty products by inhibiting the initiation and propagation of free radicals and minimizing the formation of degradation compounds [55]. Antioxidants are described as “small-amount compounds able to prevent the production of rancidity or other flavor alterations in meals owing to oxidation or greatly delaying the oxidation of readily oxidized components such as lipids [56]. Antioxidants can be classified, depending on their mode of action, into several groups. Some of them act as free radical scavengers, as these compounds can also interfere with the oxidation process as free radical terminators, and sometimes they act as metal chelators that catalyze the oxidation of lipids [45,57]. These compounds could be natural or synthetic [58].

### 4.1. Synthetic Antioxidants

**Figure 11:**
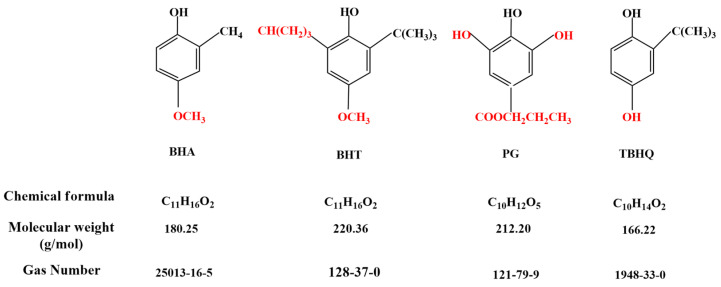
Chemical structures of butylated hydroxyanisole (BHA), propyl gallate (PG), tertiary butylhydroquinone (TBHQ), and butylated hydroxytoluene (BHT). The red color represents the differences in the chemical structure of BHA, BHT, TBHQ, and PG.

Exogenous synthetic antioxidants are compounds created through chemical processes [58]. Antioxidants can prevent or slow down food oxidation, ameliorate food stability, and prolong food storability [59]. Synthetic antioxidants can be used in foods, among which phenolic antioxidants are more frequent [60], namely BHA, BHT, PG, and TBHQ (Figure 11) [61]. Although, these phenolic compounds generally are listed as “accepted as safe”, various safety issues have been raised. Several published studies have found a link between long-term consumption of synthetic antioxidants and a variety of health concerns, including gastrointestinal disorders, skin allergies, and in some circumstances, an increase in cancer risk [56,62].

### 4.2. Natural Antioxidants

Recently, a remarkable interest to replace synthetic antioxidants with natural compounds’ antioxidant properties is an increasing trend to replace synthetic antioxidants, with natural antioxidants, which are of safety concern [63]. Thus, fruits, vegetables, grains, and MAPs are known to contain various bioactive compounds that are found to be well-associated with great antioxidant power [64]. MAPs have been used for treating human diseases since ancient memory. According to the World Health Organization (WHO), 80% of the world’s population relies on traditional medicine for their primary health care [65].

In this regard, the antioxidant properties of MAPs have been studied throughout the world as part of recent scientific developments [66]. Due to the fact that they possess a wide and diversified assortment of organic compounds that may play crucial roles and produce a specific physiological action [67]. Natural antioxidants derived from plants can be classified into three principal classes namely; phenolic compounds, vitamins, and carotenoids [68]. Among MAPs with antioxidant activity known worldwide, there are plants from several botanical families, such as *Lamiaceae* (sage, rosemary, oregano, basil, marjoram, mint, thyme, etc.), *Apiaceae* (fennel, cumin, caraway, etc.), *Zingiberaceae* (ginger, turmeric, etc.), Ginkgoaceae (ginkgo), *Asteraceae* (chamomile), and *Myrtaceae* (eucalyptus) [69].

#### 4.2.1. Phenolic Compounds

**Figure 12:**
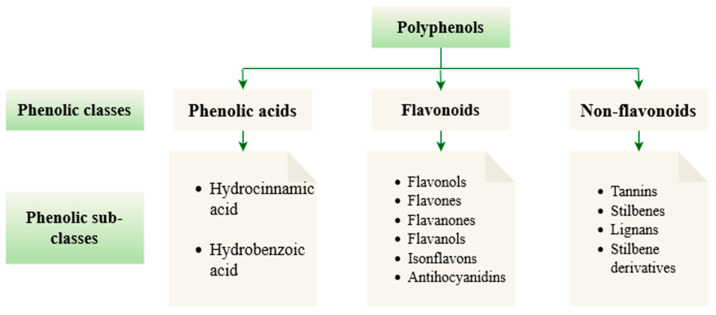
Phenolic classes and sub-classes.

Phenolic compounds are regarded as the most significant and abundant class of phytochemical compounds in the plant kingdom [70]. These phenolic compounds are ranked as secondary metabolites distributed in different plants, including roots, seeds, leaves, fruits, stems, etc. [71]. These molecules are generated by the plant to defend itself or to promote growth under unfavorable conditions [72]. Phenolic compounds contain numerous structural variants having one common structural feature, a phenol (an aromatic ring bearing at least one hydroxyl substituent), but their derivatives depend on the number of phenol subunits [73]. Based on their structure, phenolic compounds are divided into 3 major groups namely phenolic acids, flavonoids, and non-flavonoids (stilbenes and lignans), as shown in Figure 12 [74]. In addition, phenolic acids and flavonoids are the most important groups of secondary metabolites and bioactive compounds in plants [75]. The bioactivity of phenolic compounds is based on their prospective hub of natural antioxidant activity [76]. This plays a role in scavenging free radicals and reactive oxygen and nitrogen species (ROS/N), and inhibiting enzymes responsible for free radicals formation [72,77].

#### 4.2.2. Phenolic Acids

**Figure 13:**
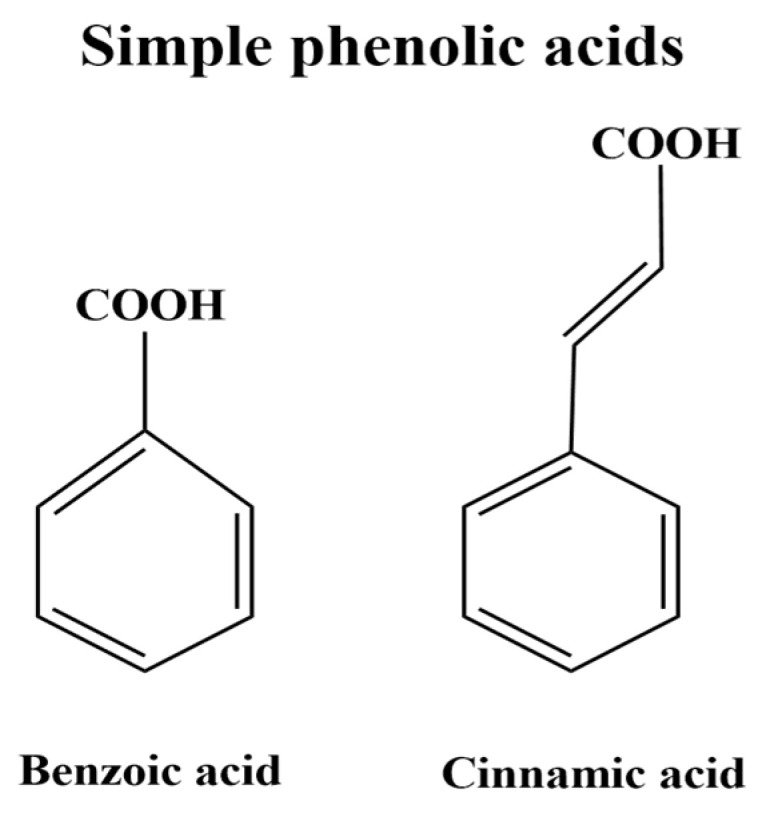
Simple phenolic acids.

The term “phenolic acids” usually the non-flavonoid molecules with a carboxylic acid group [78], which is divided mainly into two sub-groups: benzoic acid derivatives or hydroxybenzoic (C1–C6 backbone), and cinnamic acid derivatives or hydroxycinnamic (C3–C6 backbone) (Figure 13) [79]. Hydroxycinnamic acids, more common than hydroxybenzoic acids, which are found only in trace amounts (1 ppm) [80]. Ferulic, caffeic, p-coumaric, and sinapic acids are the four most prevalent hydroxycinnamic acids. The four most prevalent hydroxybenzoic acids, on the other hand, are p-hydroxybenzoic, protocatechuic, vanillic, and syringic acids [78,81].

#### 4.2.3. Flavonoid Compounds

Flavonoids (the term is derived from the Latin word “flavus”, which means yellow). It provides the coloring of flowers by producing yellow or red/blue pigmentation in shoots, leaves, buds, petals, and fruits [82]. This pigmentation is intended to attract pollinators within the flowers. Flavonoids are part of the polyphenolic compounds (They include more than 6000 among more than 8000 phenolic compounds present in plant foods), and constitute a large family of plant secondary metabolites [83]. They are physiologically active agents in plants and are becoming of great scientific interest for their health benefits [84]. The antioxidant mechanisms of flavonoids could be characterized by using direct scavenging of oxygen free radicals or excited oxygen species, chelation properties, and the inhibition of oxidative enzymes [85]. Flavonoids are classified into the following categories: flavanols, flavones, flavanones, isoflavones, and anthocyanidins, following the variety of the species, the edaphoclimatic conditions, plant tissues, growing conditions, and the degree of maturity [86].

#### 4.2.4. Non-Flavonoid Phenolic Compounds

Tannins, frequently referred to as tannic acid, naturally belong to the non-flavonoid phenolics found in many plants [87]. They are often chemically divided into two main groups: hydrolysable and condensed tannins [83]. Condensed tannins are structurally more complex and uniform than hydrolysable tannins [83]. Stilbenes, lignans, and stilbene derivatives are also a typical class of phenolic compounds found in plants. In general, all of these various molecules possess remarkable antioxidant and radical scavenging properties in plants [80,88].

There are several bioactive phenolic compounds with antioxidant functions naturally occurring in plants. Among them, antioxidant vitamins (A, C, and E), carotenoids, coenzyme Q, lycopenes, and phenolic compounds (phenolic acids, flavonoids, flavonols, anthocyanins, tannins, and lignins) [89].

- Carotenoids

Carotenoids, known also as tetraterpenoids (holding at least 40 carbons and an extensive conjugated double bond system), are natural organic non-polar pigments of yellow, orange, and red color. They are mainly produced in the plastids of medicinal plants [90]. Carotenoids are large in numbers (more than 700), six of them (*β*-carotene, *β*-cryptoxanthin, *α*-carotene, lutein, lycopene, and zeaxanthin) are the main carotenoids having antioxidant activity [91]. *β*-carotene has potential biological antioxidant activities owing to its chemical structure and its interaction with biological membranes [92]. They have the ability to scavenge radicals such as hydroxyl, peroxyl, alkoxyl, and the hydroxyperoxide anion generated from processes such as lipid peroxidation [93]. In addition to their antioxidant capacities, they can be used as food colorants [61].

- Vitamins tocopherols and tocotrienol

Vitamins are one of the most interesting lipid-soluble primary defense antioxidants, especially vitamin E [69]. Vitamin E is a generic term for all tocopherols and tocotrienols. In nature, vitamin E occurs in four tocopherol isomers (*α*-, *β*-, *γ*-, and *δ*-tocopherol) and four tocotrienol isomers (*α*-, *β*-, *γ*-, and *δ*-tocotrienol) [94]. All of these molecules have antioxidant activity as well. Although, *α*-tocopherol is chemically and biologically the most active [95]. *α*-tocopherol plays an important role in the antioxidant defense network of plants due to its superb capacity to scavenge ROS [69]. Table 2 summarizes the content of selected antioxidant compounds identified in many MAPs.

## 5. Enrichment of Oils with Natural Antioxidants

Here, VOs fortified with natural bioactive compounds are discussed. Despite their natural content of antioxidants such as tocopherols, tocotrienols, phenolic compounds, carotenoids, and sterols, oils and fats miss sufficient oxidative stability. In general, low oxidative stability is due to a major problem, known as oxidation. This induces oil quality deterioration, and it leads to health problems like colds, cancer, heart disease, mutagenicity, and other diseases [96]. In order to preserve the high nutritional value of oils and improve their oxidative stability and their shelf life, many approaches are used. Adding synthetic compounds such as BHT, BHA, PG, and TBHQ is one of the most common strategies [97]. Recently, because of the safety concerns of such synthetic compounds, there is a strong tendency to use natural bioactive compounds extracted from plants [97,98,99,100]. Along with plant MAPs extracts, essential oils are also used to prevent lipid oxidation and to flavor oily products [101,102]. Enrichment of edible VOs and other products is also practiced as functional foods, which are appreciated by consumers thanks to their benefits for health. Indeed the product is enriched with nutrients, and the flavor is ameliorated by transferring aromatic compounds into the food matrix [102]. This practice is rapidly growing worldwide [61,103]. Many plants presenting interesting sensory and phytochemical profiles such as rosemary, lavender, sage, laurel, oregano, menthe, basil, lemon, and thyme, among others, are used for this purpose. In this context, several types of flavored oils with different products (vegetables, herbs, spices, mushrooms, and fruits) are commercialized recently [103,104,105]. Nowadays, by-products from fruit and vegetable processing such as flower, kernel, peel, leaf, and roots showing a high content of bioactive compounds like phenolic acids, flavonols, anthocyanidines, flavonones, carotenoids, and glycoside are used as a principal source of natural antioxidants, to enhance oil stability [106]. Another new technique to fight against oil oxidation is the use of cereal bran extract [107]. Many studies have focused on the enrichment of edible oils with natural antioxidants.

### 5.1. Olive Oil

Olive oil is one of the most important sources of fat in the Mediterranean diet [108] and is known to be rich in unsaturated fatty acids and unsaponifiable compounds with important properties such as cardioprotective properties [109]. To preserve these properties, several studies have been carried out on olive oil oxidative stability using natural bioactive compounds from plants [106,110,111,112]. Indeed, Dairi et al. [110] reported that phenolic compounds from *Myrtus communis* L. inhibit phospholipid peroxidation in olive oil, and olive oil enriched with this plant might be a potential functional food. Blasi et al. [106], have found that adding carotenoid extract from *Lycium barbarum* L. can help to enhance the stability oxidative of extra virgin olive oil (EVOO). Hernández-Hernández et al. 2019 also found similar results using the extract of cocoa bean husk rich in theobromine and virgin olive oil jam [111]. In a similar manner, Montesano et al. 2019 demonstrated that the enrichment of EVOO with a carotenoid-rich extract from *Lycium barbarum* L. increases the nutritional value and shelf-life of added-oil, protecting EVOO natural antioxidants throughout long-term storage [112]. By-products were also used as a natural source of bioactive compounds to enrich olive oil [113]. Olive mill waste water (OMWW) was considered by several authors as the natural origin of bioactive compounds [114,115,116]. The authors of these studies concluded that crude phenolic concentrate from fresh OMWW significantly reduced the oxidation of heated oils by the *α*-tocopherol oxidation, and the formation of undesirable compounds. Dairi et al. reported that EVOO enriched with myrtle phenolic leaf extracts present better antioxidant activity than EVOO added with BHT, indeed EVOO supplemented by myrtle extract increased the loss of DPPH radical by factors of 107.8%, instead of 9.9% for BHT [110], according to the authors more research is required to explore the bioabsorption, bioavailability, and interactions between these chemicals found in myrtle enriched-EVOO, after intake. These results confirmed that OMWW produced during olive processing could be an important source of bioactive compounds. Essential oils from peppermint (*Micromeria fruticose* L.), oregano (*Origanum onites* L.), thyme (*Thymus vulgaris* L.), and laurel (*Laurus* *nobilis* L.) were also used to enrich olive oil [103]. It was proven that the main components of essential oil, like carvacrol, eucalyptol, and pulegone were transferred into olive oil samples [117].

### 5.2. Soybean Oil

The enrichment of soybean oil is extensively studied. Several studies have been previously carried out with plant extracts to prevent oxidative deterioration. Among them, olive leaves [118], some aromatic plants [119], rosemary, rambutan, fruit peel [120], grape seed [121], cocoa bean shell [122], coffee husk [123], peanut skin [124], *Cressa cretica* L. leaves [125], goji berry [126], marjoram, thyme, ginger, turmeric [127], oregano [15], watermelon [96]. It has been shown that soybean oil enriched with olive leaf extract showed lower peroxide value and a lower amount of secondary product compared to the control [118]. Phuong et al. (2019) reported that the addition of rambutan extract delayed the oxidation process, as TBHQ, and the obtained fried potatoes in the fortified oil exhibit a low level of thiobarbituric acid (TBA) [120]. The work published by Kozłowska; Gruczyńska (2018) and Yang et al. (2016) show that the addition of polyphenols, extracted from *Theobroma cacao* L., *Thymus vulgaris* L., *Rosmarinus officinalis* L., and coffee husk as antioxidants in oils effectively prevent their degradation and delay the degradation of tocopherols and polyunsaturated fatty acids. They also decrease the generation of free fatty acids, and reduce the peroxide value, but increase the antioxidant activity [15,122]. These studies suggested the potential use of these plant extracts as an effective alternative to synthetic antioxidants.

### 5.3. Sunflower Oil

Enrichment of sunflower oil also has been studied using different sources of natural antioxidants. Indeed, extracts from OMWW and olive pomace (OP) were used [128]. According to this study, extracts of these two products retarded VO oxidation during deep-frying. Similar trends were observed with extracts of spinach (*Spinacia oleracea* L.) [127,129], marjoram (*Origanum majorana* L.), thyme (*Thymus vulgaris* L.), oregano (*Origanum vulgare* L.) [15], sesame seeds [130], mango peel [131], tomato peel [132], avocado (*Persea americana* Mill. Lauraceae cv. Hass) and olive leaves [133]. The results found in these studies showed that globally the enrichment of sunflower oil with different natural antioxidants ameliorate oil stability. Indeed, spinach extract and mango peel exhibit significant effectiveness in oxidative stabilization and are beneficial for the thermal stability of sunflower oil. It has been demonstrated that the oxidative stability of sunflower oil (SFO) samples enriched with thyme and oregano extracts was enhanced compared to the control samples without the addition of herbal plant extracts and artificial antioxidants [15]. Sesame seed extracts tested by Hussain et al. (2018) could stabilize SFO and inhibits its thermal deterioration by improving its hydrolytic stability, inhibiting lipid oxidation, and reducing the loss of polyunsaturated fatty acids [130]. Jiménez et al. (2017) demonstrate that the addition of avocado (*Persea americana* Mill. Lauraceae cv. Hass) hydroalcolic extract showed a prooxidant effect, while olive (*Olea europaea* L. cv. Arbequina) hydroalcoholic leaf extracts reduce the formation of polar compounds and showed an anti-polymeric and an antioxidant effect [133].

### 5.4. Other VOs

Other VOs were also studied, flaxseed oil enriched with carotenoids from sea buckthorn pomace [134]. The main results of this study indicated that the nutritional value, quality, and stability of the enriched oil were improved. Canola oil also was enriched with extracts of different plants like basil, oregano, rosemary, and sage. According to the obtained results, methanolic oregano extract seemed to provide strong antioxidant substances that protect the polyunsaturated fatty acids [135].

Avocado, olive leaf [133], and *Teucrium polium* L. extracts were also tested on canola oil [136]. These extracts showed a good capacity to retard oil oxidation and deterioration. Rapeseed oil, corn oil, peanut oil, olive pomace oil, and grape seed oil enriched with carotenoids coming from dry tomato waste. The results obtained indicate that enriched oils show high content of carotenoids, and in some oils, the oxidative and thermal stability was improved, while in others, an increase of peroxide value and a decrease in induction time was seen [99].

## 6. MAPs Extracts for Vegetable Oils Enrichment

MAPs are considered perfect sources of natural antioxidants, such as phenolic substances, usually referred to as polyphenols, which are ubiquitous components of plants and herbs [137]. More than 8000 phenolic compounds have been reported as naturally occurring substances from plants [72]. Other types of substances in plants, such as phenolic acids, phenolic triterpenes, carotenoids, diterpenes, and flavonoids, are interesting bioactive compounds with several health properties (antioxidant, antimicrobial, antifungal, and anti-inflammatory activities) [72]. MAPs serve as an indigenous source of new compounds with therapeutic value and can also be involved in drug development [138]. Herb extracts were used as natural food additives in ancient traditions to improve sensory characteristics thanks to their health properties. The principal components found in plants correspond to four important biochemical classes namely polyphenols, terpenes, glycosides, and alkaloids [139], and many natural antioxidant compounds. These are now used in medical and pharmaceutical products as substitutes for artificial antioxidants, which are suspected to be a major cause of carcinogenesis [72]. The use of MAPs in foods is an excellent strategy to enhance the flavor and the aroma of various foods since plant extracts are rich in phytochemicals, which are of particular importance due to their health-promoting effects [119,139]. Plants extracts have been exploited to enrich VOs with natural antioxidants, as discussed in Salta et al. [140]. For instance, oregano in cottonseed oil, rosemary, and sage extracts in both palm oil and rapeseed oil, ethanolic extract of summer savory in sunflower oil, methanolic extract of tea leaves and oat extracts in cottonseed oil, and spinach powder in soybean oil. Likewise, leafy vegetable extracts (cabbage, coriander leaves, hongone, and spinach) in sunflower, as well as olive leaves, which are very studied to enrich edible oils such as olive oil [140], virgin olive oil [23], and other VOs (sunflower, soybean, palm, etc.) [140,141]. Olive leaves are rich in oleuropein a natural product of the secoiridoid group [142], known for its blood pressure-lowering effect and most abundant phenolic compounds in olive leaves [143,144]. Many studies were conducted to enrich oils with olive leaf extracts [145,146,147]. Extracts from species belonging to the *Lamiaceae* family have been reported in several studies for their antioxidative activity [148]. Rosemary was used in traditional medicine as a stimulant and mild analgesic, and it has been considered one of the most functional herbs for treating poor circulation, inflammatory diseases, headaches, and physical and mental fatigue [119]. Its extracts have been used in food preservation, as they prevent oxidation and microbial contamination [148] and also as an additive to enrich VOs, rosemary extract’s effectiveness was evaluated generally for oils during deep fat-fraying by oils such as soybean and palm oils [149,150] and also for a mixture of sunflower, soybean, and palm oils [151]. *Thymus* species are well recognized for their antispasmodic, sedative, antioxidant, and antibacterial characteristics and are frequently used in the food sector as herbal teas, aromatic, flavoring agents (condiment and spice), and medicinal plants. The preservative effect of thyme (*Thymus schimperi* R.) was evaluated on soybean oil, butter, and meat, and it was found to increase the induction time of the foods [152]. Phenolic acids, flavonoids, and phenolic monoterpenes, bioactive compounds from thyme extract were used to flavor corn refined oil enhancing its oxidative stability and antioxidant activity [153]. Oregano covers approximately 60 species known as oregano in the world [154]. High content of phenolic compounds and essential oils in oregano confers to the plant its strong antioxidant character [155], as well as other biological activities such as antimicrobial activities [156]. It was macerated in olive oil in order to improve its enrichment with antioxidants from the plant [157], and also its essential oil was used to flavor olive oil [16]. Laurel is a plant species from the *Lauraceae* family, native to the Mediterranean region, dried leaves, also known as bay leaves, and essential oil are used as a valuable spice and flavoring agent in the culinary and food industry [158,159].

Laurel essential oil effects on virgin olive oil were studied by Taoudiat et al. [104]. These authors reported that the oxidative stability of oil samples supplemented with plant extracts was improved compared to samples without the addition of herbal plant extracts. Other plants were investigated to enrich and improve oils, such as pomegranate, pistachio, walnut, savory, etc. Table 3 summarizes different plants, oils enriched, and the main results of the enrichment reported.

## 7. Extraction Methods of Antioxidants from Medicinal Plants and Enrichment of Vegetable Oils

### 7.1. Extraction Methods of Antioxidants from MAPs

**Figure 14:**
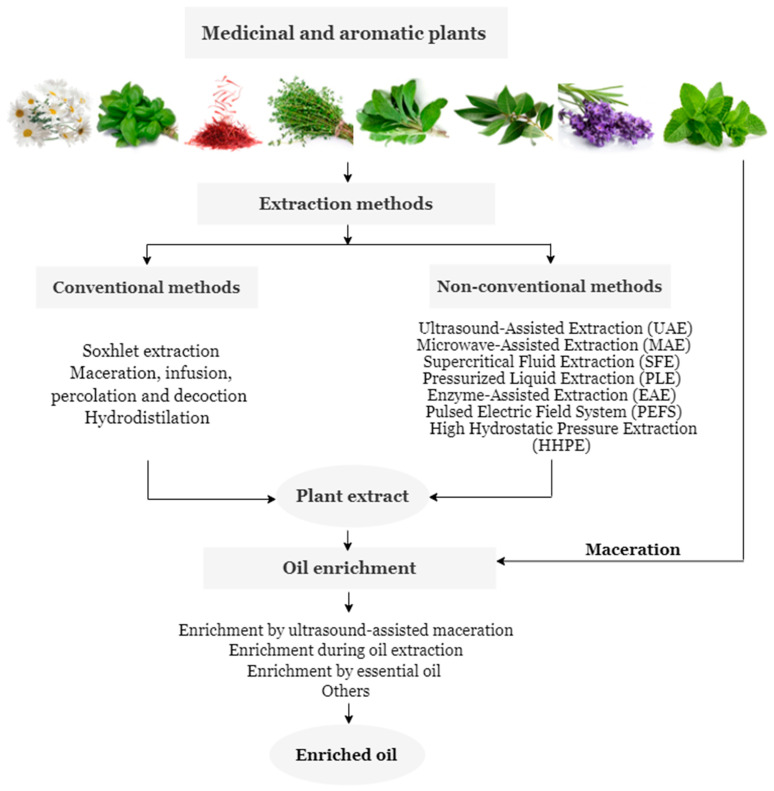
Extraction and enrichment methods of antioxidants from MAPs.

After the collection of MAPs, the extraction of the antioxidant substances represents the first step in the enrichment of oil (Figure 14) [174,175].

Extraction efficiency is influenced by several factors, such as the extraction temperature, the concentration of the extraction solvent, the extraction pH, and the extraction time, among others [176,177,178]. Solvent is one of the most critical factors, the selection of these products is based on the chemical nature and polarity of the antioxidant compounds to be extracted. The selection of solvents can be generally divided into two groups. These are polar and moderately polar solvents, just like water, methanol, ethanol, propanol, acetone, and their aqueous mixtures for the extraction of water-soluble antioxidants like phenolic compounds, flavonoids, and anthocyanins [179,180]. While familiar organic solvents, like mixtures of hexane with acetone, methanol, ethanol, or mixtures of ethyl acetate with acetone, methanol, and ethanol, have been used for the extraction of fat-soluble antioxidants, namely carotenoids [181,182]. The most commonly used extraction methods can be grouped into conventional (hot water bath, maceration, and Soxhlet extraction) [183], and non-conventional procedures [184]. The first is traditional methods, with high solvent consumption, accomplished at the level of small research or by small production companies [76]. Non-conventional methods are modern and use high energy inputs/processing capacity to improve the efficiency and/or selectivity of the extraction [185], (ultrasound, microwave, pressurized liquids, enzymatic hydrolysis, high hydrostatic pressure, supercritical fluids, and pulsed electrical field) [186].

#### 7.1.1. Conventional Extraction Methods

- Soxhlet extraction

The Soxhlet method is the most frequent method for the extraction of bioactive compounds from vegetables [187]. The Soxhlet extractor was invented by Franz von Soxhlet in 1879 [188]. The main application of this apparatus is in chemistry to dissolve weakly soluble compounds from solid matrices. It permits an unattended and unmanaged operation and efficiently recycles a slight volume of solvent to dissolve a greater volume of material [187]. Soxhlet extraction depends widely on the properties of the matrix and particle size as internal diffusion can be a limiting step of the extraction, solvents used during the Soxhlet extraction must have the necessary properties such as selectivity, solvation, distribution coefficient, density, interfacial tension, recoverability, and chemical reactivity. A co-solvent can be added to raise the polarity of the liquid phase [189]. Among the advantages of Soxhlet extraction, is that the sample is repeatedly brought into contact with a solvent. This allows the shifting of the transfer equilibrium. In addition, the system remains at a relatively high extraction temperature due to the effect of the heat applied to the distillation flask, reaching some extraction cavities. Also, there is no need for filtration after leaching [190]. However, Soxhlet extraction has a number of disadvantages, such as the long extraction time (6 h), exposure to dangerous and flammable liquid organic solvents, and the possibility of toxic emissions throughout extraction. Solvents used in the extraction system must be of high purity, which can increase the extraction price. This procedure is not considered eco-friendly and could participate in the pollution problem compared to a conventional extraction method like supercritical fluid extraction [191]. The perfect sample for Soxhlet extraction is also constrained to a dry, finely separated solid [76] as well as many factors such as solvent-to-sample ratio, temperature, and agitation speed need to be taken into account for this technique [192].

- Maceration, infusion, percolation, and decoction

Maceration requires soaking plants (coarse or powdered) in a container sealed with a solvent (called a menstruum) and left at room temperature for a minimum period of 3 days with frequent agitation until the soluble matter has dissolved [193]. The mixture is then filtered, and the solid residue is pressed to extract most of the occluded solutions, the filtered and pressed liquid obtained is mixed and separated from impurities by filtration. The final filtered liquid is evaporated and concentrated [194].

Infusion and decoction share the same principle with maceration; both are immersed in boiled or cold water [193]. In contrast, the maceration time is shorter in the case of infusion. For decoction, the sample is boiled in a given volume of water for a specified time. Decoction is only suitable for the extraction of thermostable compounds, and hard plant material, among others. Decoction is only adapted for the extraction of thermostable compounds, and hard plant materials. Decoction usually contains more fat-soluble compounds than maceration and infusion. A unique piece of equipment called a percolator is used in percolation, another extraction method with a similar basic principle [195]. Dry powdered samples are placed into the percolator, added to boiling water, and macerated for 2 h. The percolation process is usually performed at a moderate rate until the extraction is completed before evaporation. It is recommended that the extraction is completed before evaporation to obtain a concentrated extract.

- Hydro distillation

Hydro distillation is a conventional method of extracting bioactive compounds, principally essential oils from plants [196,197]. Hydrodistillation includes three main physicochemical processes namely hydrodiffusion, hydrolysis, and thermal decomposition [198]. At high extraction temperatures, some volatile constituents can be lost. This limits its use for the extraction of thermolabile substances. There are three kinds of hydrodistillation [199] called water-steam distillation, water distillation, and steam distillation. Regarding hydrodistillation, the vegetable material is first put into a compartment of the still, then sufficient water is added and then boiled. As an alternative, steam is injected directly into the plant material [200]. Although, as positive points of this kind of extraction method; it can be performed without using organic solvents and can be carried out before dehydration of the matrices used for extraction [198]. The main drawbacks of this method are the long extraction time, possible chemical changes in terpenes’ structures, and the loss of some polar molecules owing to the applied heat [198,201].

#### 7.1.2. Non-Conventional Extraction Methods

- Ultrasound-assisted extraction (UAE)

UAE has been commonly adopted in the last three decades as an important extraction efficient in pharmaceutical and food industries [202]. The mechanism is founded on the phenomenon of cavitation. The propagation of ultrasound in liquid systems is through a series of compressional and rarefaction waves, which can induce the production of cavitation bubbles within fluids [203,204]. The diameters of such bubbles expand over a few cycles until reaching a critical threshold, at which time they collapse and release a tremendous amount of energy, resulting in extraordinary temperatures (5000 K) and pressure (1000 atmospheres) at ambient temperature. During UAE, high temperature and pressure would destroy the cell walls of plant material, which facilitates the release of bioactive compounds from the plant cell walls and improve mass transport. The frequency, intensity, temperature, and duration of the ultrasound have a direct impact on the extraction frequency, and yields. In addition, solvent type and volume as well as sample characteristics such as sample particle size and moisture content are also important factors for an efficient extraction [205]. Compared to conventional methods, ultrasonic extraction has shown several advantages in terms of extraction yields and time [206].

- Microwave assisted extraction (MAE)

MAE involves three phases [207]: the detachment of solutes from the active sites from the solid matrix under elevated temperature and pressure; diffusion of the solvent through the solid matrix; and release of the solutes from the matrix into the solvent. Microwave frequency is set between 300 MHz and 300 GHz. In order to warm up quickly under microwave radiation, the solvent has to be of a high dielectric constant (which measures the efficiency at which absorbed microwave energy can be transformed into heat within a material when an electric field is applied) [208]. The advantage of this technique is the reduction in extraction time and solvent volume compared to the conventional method (maceration and Soxhlet extraction). By using appropriate conditions, in order to avoid thermal degradation, better recoveries have been observed in the MAE method [209].

This approach, however, is restricted to small phenolics such as phenolic acids (gallic acid and ellagic acid), quercetin, isoflavin, and trans-resveratrol thanks to their stability at microwave heating conditions of up to 100 °C for 20 min. Additional cycles of MAE resulted in a drastic decrease in the yield of phenolics and flavonoids. The yield of phenolics and flavanones decreased, mainly owing to the oxidation of the compounds [210]. Tannins and anthocyanins may not be suitable for MAE, as they are potentially subject to high-temperature degradation [211].

- Supercritical fluid extraction (SFE)

SFE, as an environmentally sustainable technique, has been widely used recently [212]. Over the critical pressure and temperature, the solvent may enter the supercritical state, which has both liquid-like (solvent power, negligible surface tension) and gas-like (high diffusivity and low viscosity) characteristics [212,213]. SFE uses solvents at temperatures and pressures beyond their critical points. Compared to normal liquids, supercritical liquid fluids can achieve improved transport qualities, which diffuse rapidly via solid materials, and thus achieve quicker extraction rates [200]. The strength of supercritical solvents can be easily modified by changing the pressure, temperature, or by adding modifiers to reduce the extraction [214].

- Pressurized liquid extraction (PLE)

PLE is based on the use of solvents at elevated pressure and temperature to extract the desired component from the different matrices [174,215]. By increasing pressure, the temperature of the solvent in the liquid state may be higher than its boiling point at normal temperature, which could increase mass transfer and improves the solubility of analytes. By elevating pressure, the temperature of the solvent in the liquid state may be higher than its boiling point at normal temperature, which can increase mass transfer and improve the solubility of analytes. This extraction method may be performed over a temperature range of 21 to 200 °C and a pressure range of 35 to 200 bars [174]. If water is used as a solvent, PLE is also known as subcritical water extraction (SWE) [216]. As the water temperature is increased to 200–250 °C in SWE, it may be kept in a liquid state, whilst the dielectric constant (ε) of water is reduced from 80 to 25, which is similar to the dielectric constant of some organic solvents like methanol or ethanol [174,217].

- Enzyme-assisted extraction (EAE)

EAE is a potentially green extraction method due to the soft extraction conditions and its eco-friendship [218].

Enzymes are characterized by their high specificity and efficiency. They have the ability to degrade compositions and destroy the structural continuity of the plant cell wall, this latter promotes the liberation of bioactive constituents. Among the used enzymes, in this extraction method, are hemicellulase, cellulase, pectinase, and *β*-glucosidase. These enzymes could be extracted from different sources such as fungi, bacteria, fruit and vegetable extracts, or animal organs [183,218]. Several studies have demonstrated that EAEs improve the extraction performance of antioxidants, especially phenolics, flavonoids, and carotenoids [219,220,221].

- High hydrostatic pressure extraction (HHPE)

HHPE is for very high cold isostatic hydraulic pressure ranging from 100 to 800 MPa and more [222]. HHPE is a new approach used for active constituents extracted from natural biomaterials. The advantage of this method is to improve mass exchange ratios, boosting cell permeability, as well as the diffusion of secondary metabolites in accordance with changes in phase transitions [223]. HHPE has been applied for the extraction of ginsenosides from Korean red ginseng [224], flavonoids from propolis [225], polyphenols from green tea leaves [186], and anthocyanins from grape by-products [223]. The use of HHPE has been shown to be very efficient, compared to conventional or other novel extraction methods by offering high extraction efficiencies and high extraction selectivity, as well as shorter time (1 min for most studies) and requiring less energy [186].

- Pulsed electric field system (PEFS)

PEFS is a technique founded on the use of short-period pulses of high electrical field intensity (0.1–50 kV/cm) at ambient temperature [226]. The goal of PEFS applications is to make cell membranes permeable to improve the transfer of components from inside the cells [227]. Electrical fields of a few to hundred microseconds are able to intimate the formation of pores in the cell membrane, called also “electroporation”. On this basis, subsequent extraction of bioactive molecules can be performed [228]. Different investigations and advantages of pulsed electric field treatment have been found to enhance the extraction of bioactive compounds (antioxidants, tocopherols, polyphenols, and phytosterols) from various fruits, vegetables, and agricultural wastes [229,230]. Table 4 presents some examples of extraction methods for natural antioxidants.

### 7.2. Enrichment Methods for VOs with MAPs

The enrichment of edible VOs with antioxidant substances can be achieved in different ways [204,240].

- Enrichment by natural maceration

One of the methods that can be carried out is enrichment by maceration is an old and easy-to-carry-out principle [17]. It allows extraction of liposoluble active ingredients by simple pressing, by mixing plant extracts in a fatty substance that acts as a natural solvent [241]. Valerija et al. have used it to enrich refined rapeseed oil with phenols and chlorophylls from olive leaves. Healthy leaves were sampled from the olive branches and washed in distilled water four times, three forms (whole, cut, and crushed) of fresh or dried olive leaves were prepared for maceration in oil ovens. The maximum total phenolics (220.4 ± 5.3 mg/kg) was achieved in VOs with fresh whole leaves after seven days of maceration, but the conversion of chlorophylls to oils was most effective when crushed and steam-bleached leaves were macerated for 28 days (79.10 ± 1.14 mg/kg) [242].

- Enrichment by ultrasound-assisted maceration

Recently, new techniques have been developed for more efficiency regarding oil enrichment [157]. Namely, the enrichment of oils using ultrasounds; this method has shown good extraction results since it allows penetration and mass transfer [240]. Thanks to the cavitation principle that fosters the formation of tiny bubbles subjected to rapid adiabatic compression and expansion [190]. Achat et al. [190] adopted the ultrasonic maceration method to enrich olive oil with phenolic compounds from olive leaves under the following conditions: temperature of 16 °C, ultrasonic power of 60 W, and sonication time of 45 min.

- Enrichment during oil extraction

In the same context, the study of Sanmartin et al. proposed a green, efficient, and innovative enrichment procedure. In the experimental conditions adopted, citrus and olive leaves are crushed and cryo-macerated with the olives during the extraction of oil. A higher antioxidant content was calculated in the enriched olive oils compared to the control sample, and a high concentration of oleuropein was detected in the olive oil extracted in the presence of the olive leaf (+50% in the olive oil). The organoleptic profiles of the enriched olive oils were also profitably improved in terms of overall pleasantness and odor complexity, compared to the control [48].

- Enrichment with essential oil

Another technique aims at enriching VOs with an essential oil obtained from plants, as was done by Asensio et al. [16]. To this end, olive oil was flavored with oregano essential oils (OEO). Olive oil samples were spiked with 0.05% OEO and stored under dark and light conditions for 126 days. Samples with OEO showed low values of lipid oxidation indicators (UV absorption coefficients: K232, K269, peroxide value, and anisidine value), especially in the dark. Olive oil with OEO in dark displayed a low peroxide value (18.71 mEqO_2_ kg^−1^) [16].

- Other techniques

Meanwhile, Medina et al. [147] have enriched various refined oils with phenolic extracts of olive leaves and olive pomace, by applying an alternative enrichment technique consisting of first preparing ethanolic extracts of olive leaves and pomace, adding them to refined oils, and finally evaporating the ethanol from the two-phase system. A significant improvement in the quality and stability parameters of the enriched oils was recorded [147]. Comparable results were found by Kozłowska and Gruczyńska [15] who evaluated the oxidative stability of sunflower and soybean oils enriched with plant extracts (marjoram, thyme, and oregano) using the same procedure.

On the other hand, Şahin et al. investigated the enrichment of corn oil with polyphenols by adding olive and lemon balm leaves extracts. After evaporation of the solvent in the extraction step, the extracts were dried and then partially dissolved in corn oil by a solid-liquid extraction method. The total phenolic content has been improved by 9.5 and 2.5 times compared to pure corn oil, and the antioxidant activity of the oil enriched with olive and lemon balm leaves extracts was found to be almost 14 and 6 times higher, respectively, than those of the untreated oil, and therefore the improved oil stability (18%) [161].

## 8. Conclusions

Here, we highlighted the use of aromatic and medicinal plant extracts to improve the nutritional value and oxidative stability of vegetable oils. The bibliographic analysis carried out for this paper revealed a significant number of articles describing the importance of antioxidants in protecting edible oils against autoxidation. Edible VOs enriched with natural antioxidants extracted from MAPs have also been reported to have considerable antioxidant activity and thermal stability. The utilization of natural antioxidants extracted with durable and sustainable techniques from MAPs is an innovative way of achieving a circular economy and responding to consumer needs for natural and healthier foods. However, it is important to choose the appropriate extraction and enrichment methods, and subsequently the most effective concentrations for a functional food design. Moreover, further works concerning the bioactive compounds of extracts showed significant effects on the stability of vegetable oils should be investigated, determining the mechanisms related to their effects.

## Figures and Tables

**Table 1:** Most productive journals in the enrichment of VOs with natural antioxidants.

Journal	Publisher	Total Documents	Total Citations	Average Citation per Journal
Journal of Food Science	Wiley-Blackwell	6	112	18.67
Journal of Agricultural and Food Chemistry	American Chemical Society	4	155	38.75
Food Chemistry	Elsevier	3	328	109.33
International Journal of Molecular Sciences	MDPI *	3	121	40.33
Journal of Food Processing and Preservation	Wiley-Blackwell	3	24	8.00
Journal of Food Science and Technology	Springer Nature	3	34	11.33
European Journal of Lipid Science and Technology	Wiley-Blackwell	2	11	5.50
Food Research International	Elsevier	2	116	58.00
Industrial Crops and Products	Elsevier	2	57	28.50
Innovative Food Science and Emerging Technologies	Elsevier	2	74	37.00
Molecules	MDPI *	2	12	6.00

* MDPI: Multidisciplinary Digital Publishing Institute (MDPI).

**Table 2:** Phenolic compounds and flavonoids concentration identified in MAPs.

Plants	Compounds	Concentrations	Reference
*Glycyrrhiza glabra* L.	Phenolics and terpenoids	4.94 ± 0.43 g/100 g	[64]
*Rauwolfia serpentina* (L.) Benth	Alkaloids	2.06 ± 0.11 g/100 g	[64]
*Geranium sanguineum* L.	Catechins and proantho-cyanidines	2.1 mg/kg	[65]
*Dracocephalum moldavica* L.	Rosmarinic acid	247.95 ± 24.78 mg/g	[65]
*Dracocephalum moldavica* L.	Chlorogenic acid	1.46 ± 2.76 mg/g	
*Dracocephalum moldavica* L.	Pigenin-7-O-glucoside	6.55 ± 2.20 mg/g	
*Ficus microcarpa* L. fil	Protocatechuic acid	6.60 ± 0.20 mg/g dry extract	[65]
	Catechol	11.1 ± 0.00 mg/g dry extract	
	P-vinylguaiacol	4.40 ± 0.07 mg/g dry extract	
	Vanillin	4.27 ± 0.02 mg/g extract	
	Syringaldehyde	8.96 ± 0.29 mg/g extract	
*Hibiscus cannabinus* L.	Flavanoid content	82.11 mg/g extract	[66]
*Trigonella arabica* Delile.	Tannin content	2 ± 0.47 mg TA/g	[67]
*Trigonella berythea* Boiss. & Blanche		9 ± 0.47 mg TA/g	[67]
*Origanum vulgare* L.ssp. hirtum (Link)	Rosmarinic acid	116.7 g/kg dry extract	[80]
	Carvacrol	94.6 ± 21.16 g/kg dry extract	
*Origanum vulgare* L.	Rosmarinic acid	12.88 mg/g plant	[80,88]
	Chlorogenic acid	2.10 mg/g plant	
	Hyperoside	1.05 mg/g dry extract	
	Isoquercitrin	0.71 mg/g dry extract	
	Salvianolic acid A	66.4 ± 1.7 g/kg dry extract	[80]
*Satureja thymbra* L.	Cafeic acid	2.69 ± 0.1 g/kg dry extract	
	Taxifolin	4.28 ± 0.03 g/kg dry extract	[80]
*Thymus capitatus* (L.) Hoffm. and Link	Eriodictyo	2.36 ± 0.12 g/kg dry extract.	

**Table 3:** Most plants used for vegetable oils enrichment.

Plant Common Name	Scientific Name	Part Used	Oil Enriched	Concentration	Main Results	Reference
Olive tree	*Olea europaea* L.	Leaves	Sunflower oil	200 mg of TPC of methanol extract/kg of oil	Increase in TPC (nd-155 mg CAE), AA (282–504 mg TE) and OS (1.3–2 h).	[140]
				400–2400 ppm (juice)	Improvement of oil quality during heating process (viscosity, acid value, peroxide value).	[160]
			Corn oil	1000–1500 ppm (ethanol-water extract)	TPC (18.00 ± 0.09–172.57 ± 0.53 ppm), AA (1.72–23.85%), TCC (nd-3.64 ± 0.01 mg β carotene/kg-oil)	[161]
			Refined olive oil	plus 500 µL of extract (ethanol-extract)	Increase in total polyphenol area (from 0.1 ± 0.1 to 22.5 ± 0.4)	[141]
			Olive oil	1 g of milled leaves/10 mL of oil	Enrichement of oil with 14.45 ± 3.35 µg/mL of Oleuropein.	[146]
				20 kg of fruits with 5 L of water olive leaves extract (OLE)	OLE enhanced TPC about 10% (150.9 ± 11.3 μg GAE/g of oil)	[162]
				3% of leaves extract (methanol extract)	Increase in TPC and antioxidant activity	[163]
			Refined olive oil	400 ppm of chlorophyll pigment (ethanol extract)	Incresase in chlorophyll content of oil enriched (1.46 ± 0.08 to 4.13 ± 0.02 mg/kg)	[145]
			Refined Soybean oil Palm oil Maize oil Rapeseed oil Extra virgin oil olive oil	200 and 400 μg/mL of phenols (ethanol extract)	Additional stability and impovement quality parameters and transfert of oleuropein to target oils	[147]
Rosemary	*Rosmarinus officinalis* L.	Leaves	Chia oil	1000 mg/kg (ethanol-eau extract)	Improvement of the oxidative stability From an induction period of 0.43 ± 0.01 h to 1.30 ± 0.06 h	[164]
			Flax oil	1000 mg/kg (ethanol-eau extract)	Improvement of the oxidative stability From an induction period of 0.37 ± 0.02 h to 1.17 ± 0.20 h	
			Hemp oil	20 mg of rosemary leaves extract (ethanol, methanol; acetone; ether)/100 g of oil	Improvement of the oxidative stability From a peroxide value of 105.93 ± 0.12 mEqO_2_/kg to 98.70 ± 0.50 mEqO_2_/kg for enriched hemp oil	[165]
			Sunflower and soybean mixture oil	Ethanol extract (Concentration not determined)-	Improvement of the oxidative stability Enriched oils keeps the lower peroxide value, acidity and saturated fatty acids	[151]
		Commercial rosemary extract with a very high carnosic acid content of 70%	Soybean oil	400 mg/kg of commercial rosemary extract with a very high carnosic acid content of 70%	Improvement of the oxidative stability From an induction period of 2.2 ± 0.22 h to 3.4 ± 0.18 h From a peroxide value of 23.72 ± 0.51 mEqO_2_/kg 17.32 ± 0.15 mEqO_2_/kg	[117]
			Cotton oil		Improvement of the oxidative stability From an induction period of 1.88 ± 0.2 h to 3.35 ± 0.15 h From a peroxide value of 19.47 ± 0.18 mEqO_2_/kg 16.53 ± 0.24 mEqO_2_/kg	
			Rice bran oil		Improvement of the oxidative stability From an induction period of 3.83 ± 0.07 h 6.22 ± 0.21 h From a peroxide value of 29.45 ± 0.61 mEqO_2_/kg 19.00 ± 0.19 mEqO_2_/kg	
		Leaves	Virgin olive oil	5% (*w*/*v*) of leaves/oil	Increase in the content of free fatty acids from 0.42 ± 0.01 g/100 g to 0.57 ± 0.02 g/100 g From an induction period of 3.75 h to 4.5 h	[166]
Oregano	*Origanum vulgare* L.	Leaves	Soybean oil	0.01%, 0.03% and 0.07% (Ethanol/water (7/3) extract)	Improvement of oxidative stability (*t*_ON_/°C = 155.22 ± 0.42 at 0.01% to 159.35 ± 0.69 at 0.07%)	[15]
			Sunflower oil	400 ppm (Aqueous–ethanolic extract)	Increase in antioxidant activity	[167]
			Extra virgin olive oil	10, 20 and 40 g of extract obtained by infusion/L	Improvement of oxidative stability	[14]
Laurel	*Laurus nobilis* L.	Essential oil	Extra-virgin Olive oil	0.01% of essential oil (volume of essential oil/volume of extra virgin olive oil)	Improvement of oxidative stability	[104]
Thyme	*Thymus schimperi* R.	Leaves and flowers	Soybean oil	0.1 and 0.2% (Ethanol extract)	Increase in the induction time of soybean oil from 1.92 to 3.25 h Increase in the protection factor from 1.00 ± 0.042 to 1.69 ± 0.010	[152]
	*Thymus vulgaris* L.		Soybean oil	0.01%, 0.03% and 0.07%	Improvement of the oxidative stability (From *t*_ON_ of 145.86 ± 0.47 to 156.86 ± 0.84 at 0.07%)	[15]
			Refined corn oil	5 g/40 mL of oil	Increase in the TPC from 23.63 mg/100 g to 53.99 mg/100 g Increase in antioxidant activity from 100.66 mg GAE/ 100 g to 185.22 mg GAE/100 g	[168]
Basil	*Ocimum basilicum* L.	Leaves	Olive oil	150 g of basil leaves/1 L of oil	Increase in Linalool and Eugenol ercentages	[169]
			Soybean oil	3000 mg of basil ethanol extract/kg of oil	Improvement of oxidative stability	[170]
			Sunflower oil	100 ppm and 400 ppm ofaqueous–ethanolic extract	Increase in antioxidant activity at 400 ppm	[167]
Pomegranate	*Punica granatum* L.	Juice	Pomegranate seed oil	(0%, 25%, 50%, 75%, and 100%) of juice	TPC (0.72–6.4 mg gallic acid/g) at 100%	[171]
Pistachio	*Pistacia* spp.	Kernels	Virgin pistachio oil Walnut oil	-	TPC = 407 ± 7 mg/kg gallic acid DPPH = 13 ± 1 44 ± 3 mmol/kg Trolox Improvement of the oxidative stability	[172]
Walnut	*Juglans nigra* L.		Virgin pistachio oil Walnut oil	-	TPC = 339 ± 6 mg/kg gallic acid DPPH = 44 ± 3 mmol/kg Trolox Improvement of the oxidative stability	[172]
Peppermint	*Mentha piperita* L.	Leaves	Refined rapeseed and Sunflower oils	100 ppm–400 ppm of aqueous–ethanolic extract	Decreasing in DPPH antioxidant activity for rapeseed oil Increase in DPPH antioxidant activity for sunflower oil	[167]
Savory	*Satureja thymbra* L.		Refined rapeseed and Sunflower oils		Higher antioxidant activity at 400 ppm	[167]
Sage	*Salvia officinalis* L.		Sunflower oil		Higher antioxidant activity at 100 ppm than oil supplemented by BHA	[167]
Catnip	*Nepeta cataria* L.	Leaves and flowering parts	Sunflower oil	600 and 1200 ppm of acetone extract	Increase the production of hydroperoxides for both concentrations An increase in the formation of hexanal for 600 ppm and a decrease for 1200 ppm	[173]
Hyssop	*Hyssopus officinalis* L.				An increase in the production of hydroperoxides for 600 ppm and a decrease for 1200 ppm A decrease in the formation of hexanal for both 600 ppm and 1200 ppm	
Lemon balm	*Melissa officinalis* L.				Decrease the production of hydroperoxides for both 600 and 1200 ppm A decrease in the formation of hexanal for both 600 ppm and 1200 ppm	
Pepper	*Capsicum annuum* L.	-	Extra virgin olive oil	10, 20 and 40 g of powder/L of oil	Improvement of oxidative stability	[14]
Garlic	*Allium sativum* L.	-		20, 30 and 40 g of powder/of oil	Improvement of oxidative stability	[14]

AA = Antioxidant activity, BHA = Butylated hydroxyanisole, CAE = Catechin acid equivalent, DPPH = 2,2-diphenyl-1-picrylhydrazyl, GAE = Galic acid equivalent, TCC = Total carotenoid content, TE = Trolox equivalent, TPC = Total phenolic content, TON = Thermoxidation onset temperature OLE = Olive leaves extract, OS = Oxidative stability.

**Table 4:** Examples of extraction methods of natural antioxidants.

Extraction Method	Plant	Main Compounds	Main Results (Extract)	Reference
Soxhlet extraction	Spearmint (*Mentha spicata* L.)	Flavonoids	Catechins = 0.144 mg/g	[187]
Maceration	Summer savory (*Satureja hortensis* L.)	Phenols Flavonoids Anthocyanins	TPC = 125.34 ± 0.13 mg GAE/g TFC = 16.27 ± 0.34 mg RU/g TAC = 115.21 ± 0.95 mg C3G/g	[231]
Micro-waves assisted extraction	*Pistacia* leaves (*Pistacia lentiscus* L.)	Phenols	TPC = 149.39 ± 8.11 mg GAE/g	[232]
Ultrasound assisted extraction	Rosemary leaves (*Rosmarinus officinalis* L.)	Phenols	TPC = 2040 ± 40 ppm GAE TPC = 35.0 mg GAE/g	[233,234]
Supercritical Fluid extraction	Rosemary (*Rosmarinus officinalis* L.)	Carnosol Carnosic acid	EC_50_ (DPPH) = 0.23 mg/mL	[235,236]
Pressurized liquid extraction	Spinach *(Spinacia oleracea* L.)	Tocopherols Tocotrienols	*α*-T = 284 ± 13 μg/kg *β*-T = 8 ± 0.1 μg/kg *γ*-T = 83 ± 3 μg/kg	[236]
High hydrostatic pressure extraction	Green tea (*Camellia sinensis* L.) leaves	Phenols	Yield of polyphenols at 4 min = 30.7 ± 0.8%	[237]
Pulsed electric field	Norway spruce (*Picea abies* L.)	Phenols	TPC = 8.52 g GAE/100 g	[238]
Enzyme-assisted extraction	Stevia (*Stevia rebaudiana* (Bert.)	Flavonoids	Catechins = 89–102 g/100 g	[239]

GAE = galic acid equivalent, EC = effective concentration, TPC = total phenolic content, TFC = total flavonoid content, TAC = total antioxidant capacity, DPPH = 2,2-diphenyl-1-picrylhydrazyl, *α*-T, *β*-T, and *γ*-T = *α*-, *β*-, and *γ*-tocopherols.

## Data Availability

Not applicable.
